# Autophagy inhibition plays the synergetic killing roles with radiation in the multi-drug resistant SKVCR ovarian cancer cells

**DOI:** 10.1186/1748-717X-7-213

**Published:** 2012-12-17

**Authors:** Bing Liang, Dejuan Kong, Yang Liu, Nan Liang, Mengzi He, Shumei Ma, Xiaodong Liu

**Affiliations:** 1 Key Laboratory of Radiobiology (Ministry of Health), School of Public Health, Jilin University, 1163 Xinmin Street, Changchun, Jilin 130021, China; 2 Key Laboratory of Radiobiology (Ministry of Health), School of Public Health, Jilin University, Department of Radiology and Radiation Oncology, China-Japan Union Hospital, Changchun 130021, China

**Keywords:** Autophagy, Radiosensitivity, Multidrug resistance, Ovarian cancer, Apoptosis

## Abstract

**Purpose:**

Autophagy has attracted attentions as a novel mechanism for tumor development. In this study Human ovarian carcinoma cell line SKOV3 and multidrug-resistant phenotype SKVCR cells were used and the roles of autophagy in radiation-induced cell death were analyzed.

**Methods and materials:**

Cell viability was examined by colony formation and cell counting kit-8 (CCK-8) assay, 3MA and ZVAD were used to block autophagy and apoptosis, respectively. Quantitative real-time PCR was used to detect mRNA level and Western blot was used to detect protein expression, monodansylcadaverine (MDC) staining and flow cytometery were used for autophagy, apoptosis and cell cycle dynamics, respectively.

**Results:**

(1) The radiosensitivity exhibited differently in SKOV3 and SKVCR cells (SKOV3: D0=3.37, SKVCR: D0= 4.18); compared with SKOV3 the constitutive expression of MAPLC3 in SKVCR was higher, but no change of Caspase-3 and cleaved Caspase-3. (2) The ionizing radiation (IR)- induced apoptosis and autophagy were significant in both cells (P<0.05); inhibition of apoptosis with ZVAD showed no impact on survival of SKOV3 and SKVCR cells after radiation, while inhibition of autophagy significantly decreased viability in SKVCR cells, for SKVO3 cells only low level of radiation (2 Gy and 4 Gy) could decrease the viability(P<0.05). (3) ZVAD inhibited apoptosis and autophagy in both cells, 3MA inhibit apoptosis in SKOV3, and promote apoptosis in SKVCR, together with inhibition of autophagy. (4) G2/M arrest was induced by radiation in both cells; the accumulation of G2/M was more significant in SKOV3, 3MA attenuated the radiation-induced S phase delay in SKVCR.

**Conclusion:**

IR-induced autophagy provides a self-protective mechanism against radiotherapy in SKVCR cells, the use of autophagy inhibitor, 3MA, increases the killing effects of radiation by inhibiting autophagy and radiation- induced S phase delay, also by the increase of apoptosis, which suggests a better therapeutic strategy in drug- resistant SKVCR ovarian cancer cells.

## Introduction

Epithelial ovarian cancer(EOC)is the most lethal Gynecologic malignancy, with 21,990 estimated new cases and 15, 460 deaths in USA in 2011[1]. Platinum/paclitaxel-based chemotherapy is the current standard of treatment after surgical staging and resection of abdominal and pelvic cancers. Despite the advances in chemotherapy, the prognosis still remains poor since many patients develop abdominal or pelvic recurrence that is resistant to further chemotherapy. Radiotherapy has been shown to produce a response in chemo-resistant ovarian cancers, and may offer the possibility of improved tumor control[2].

Tumor cells have the capacity to respond to chemotherapy and radiation through multiple growth arrest and cell death pathways [3-5]. Various modes of cell death have been known, such as necrosis, apoptosis and autophagic cell death [6-8].

Apoptosis, the type I programmed cell death, has been widely investigated under different circumstance including radiotherapy and the one of the most important strategies for cancer treatment over the past decades. However, apoptosis is not the predominant form of cell death as we have predicted, accounting for only 20% of cases[9]. While, autophagy, the type II programmed cell death, has been recently reported to play roles in the development of cancer. Autophagy is an evolutionarily conserved catabolic process for the degradation and recycling of cytosolic, long-lived, or aggregated proteins, and excess or defective organelles, and is primarily a response to the stress of irradiation [10], chemotherapeutic agents [7], starvation [11,12], growth factors withdrawal [13], hypoxia or viral infection [14-17]. Dual roles of autophagy have been reported, promotion of cell survival or leading to cell death [18,19]. Therefore, autophagy is considered to be a double-edged sword in the process of tumor development. Elucidation of autophagy roles in treatment responsiveness at different stages of cancer progression is a complex and challenging task. Better understanding of autophagy regulation and its impact on treatment outcomes will potentially provide novel therapeutic targets in cancer.

In this study, the human ovarian cancer cell line SKOV3 and multidrug-resistant phenotype SKVCR cells were used and the killing effect of different radiation formulae was assessed, the inhibitors of autophagy and apoptosis were used to explore the synergetic effect and the potential mechanism. These results will contribute to improve the treatment efficacy for radiation-resistant MDR phenotype ovarian cancer and bring new insights for cancer development.

## Materials and methods

### Cell culture

Human ovarian carcinoma cell lines SKOV3 and multidrug-resistant phenotype SKVCR cells were obtained from British Columbia Cancer Research Centre, Vancouver, BC, Canada. Cells were maintained in α-MEM with 10% fetal bovine serum and 100 U/ml of penicillin/streptomycin, at 37°C in humidified atmosphere containing 5% CO2. SKVCR cells were cultured in α-MEM medium containing 2.0μg/ml vincristine (VCR), to maintain the drug-resistant phenotype.

### Irradiation and chemicals

Different radiation formulae were introduced as follows: (A) 0Gy: no treatment was given; (B) 2Gy×5: 2 Gy in every day for 5 times; (C) 1Gy ×2 ×5: 2 Gy (1 Gy 2 times a day, 4 h interval) in every day for 5 times; (D) 10Gy×1: 10 Gy for 1 time. ZVAD (20μM, Enzo, USA) and 3MA(2.5 mM or 5 mM Sigma-Aldrich Inc, USA) were used as inhibitor of apoptosis and autophagy, respectively. These agents were administered into cells 2 h before radiation.X-ray irradiation was performed by using 180-KVp X-ray generator (Model XSZ-Z20/20, China) at a dose rate of 0.40Gy/min.

### CCK-8 assay

Radiosensitivity of SKOV3 and SKVCR was assayed by using a Cell Counting kit-8 (CCK-8, Dojin Laboratories, Kumamoto, Japan). 1×10^4^ cells/well were seeded in 96-well plates containing complete medium and incubated for 24 h followed by different doses of ionizing radiation for 48h. 10 μl of the CCK-8 solution was added to each well and went on with incubation for 2 h in incubator. The absorbance was measured at 450 nm using a microplate reader (Synergy HT, Bio-Tek, USA).

### Colony formation assay

After the treatment of radiation, cells were plated into 60mm petri dishes using standard culture media. Two weeks later, cells were fixed with 4% formaldehyde, stained with crystal violet and colonies containing more that 50 cells were counted and normalized to their corresponding non-irradiated control. The surviving fraction for a given treatment was calculated as the plating efficiency of the irradiated samples relative to that of the sham-irradiated ones. For each dose level in four groups, three independent experiments were done. Multi-target click model of GraphPad Prism 5.0 (Systat Software, USA) was used to fit cell survival curves. The dose quasithreshold (Dq) and mean lethal dose (D0) were calculated.

### Quantitative real-time PCR

Total RNA was isolated using RNAiso Plus (Takara Co. Japan) according to the manufactures’ instructions. Quality and quantity of RNA was analyzed by measuring the A260/A280 ratio with ultraviolet spectrophotometry. Reverse transcription was performed with 2 μg of total RNA using PrimeScript Rtreagent Kit (Takara Co, Japan). Then each sample was analyzed by quantitative real-time PCR (qPCR) (Stratagene MX3000P, Japan) in the SYBR Premix Ex TapII (Takara Co, Japan),setting the cycles as follows:10s/95°C PCR initial activation step; 40 cycles of denaturation for 20 s/95°C and annealing step for 20 s/60°C. The change in mRNA levels was determined by the formula 2−(ΔΔCT), where ΔCT is the value from the threshold cycle (CT) of the treated sample subtracted from the CT value of untreated or zero time-point control sample. The relative amount of mRNA in the sample was normalized to GAPDH mRNA. The primers for genes were as follows: MAPLC3II forward: 5^′^-CCTAGAA GGCGCTTACAGCT-3^′^ and reverse 5^′^-GGGACAATTTCATCCCGAAC-3^′^; Caspase 3 forward: 5^′^-TTCAGGCCTGCCGTGGTACA −3 and reverse: 5^′^-CCAAGAATAATA ACCAGGTGCT-3;GAPDH forward: 5^′^-GCACCGTCA A GGCTGAGAAC-3^′^ and reverse 5^′^- TGGTGA AGA CG CCAGTGGA-3^′^.

### Western blot analysis

After the indicated treatment, 40 μg of total protein was separated by SDS-PAGE, using a gradient gel [(10–12%), Bio-Rad Laboratories], transferred to nitrocellulose membrane, and analyzed by immunoblotting using the chemiluminescence (Santa Cruz, CA, USA). The primary antibodies used were Caspase 3 (Cell Signaling, Beverly, MA, USA,1:500), MAPLC3 (Cell Signaling, Beverly, MA, USA,1:500) or GAPDH (Santa Cruz, CA, USA,1:1000), peroxidase-conjugated anti-mouse IgG or peroxidase-conjugated anti-rabbit IgG. (Santa Cruz, CA, USA, 1:1000). The intensity of protein bands were quantified using image j software and the ratio of specific band to control was analyzed.

### MDC assay for visualization of autophagic vacuoles

Cells were seeded on cover slips over night followed by treatment with different doses of radiation, 48h later autophagic vacuoles were labeled with monodansylcadaverine (MDC) by incubating cells with 50 μM MDC in α-MEM at 37°C for 1 h. After incubation, cells were washed with PBS and fixed with a solution of 4% paraformaldehyde for 20 min. Autophagic vacuoles were examined using a fluorescence microscopy (Olympus, XSZ-D2).

### Flowcytometry analysis

Cells were collected 24 h after radiation and 5 × 10^5^ of cells were used for each sample. For cell cycle distribution analysis, cells were stained with RNase -containing PI (propidium iodide) solution. For apoptosis detection, cells were stained with PI and FITC labeled Annexin-V. Stained cells were detected by Flow Cytometry (BD Biosystems, USA) and data were analyzed with CellQuest (BD Biosciences) and FlowJo softwares (Tree Star Inc.).

### Statistical analysis

All experiments were performed thrice and the data were expressed as means ± SD. The difference between two mean values was evaluated by using the Student’s t -test and considered to be statistically significant when P < 0.05.

## Results

### The radiosensitivity in human ovarian carcinoma SKOV3 and multidrug-resistant phenotype SKVCR cells

**Figure 1:**
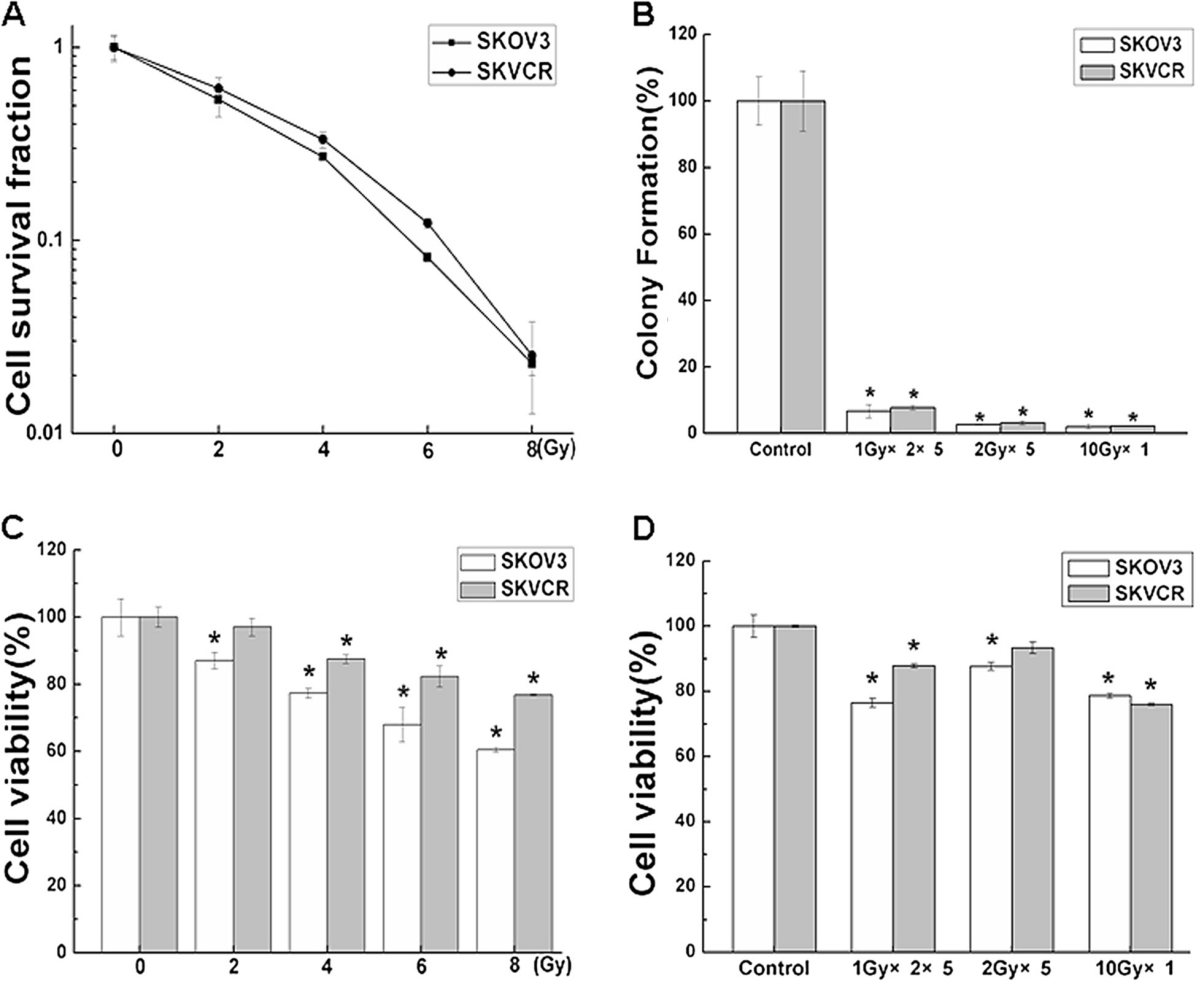
**The radiosensitivity in Human ovarian carcinoma cell lines SKOV3 and multidrug-resistant phenotype SKVCR cells. **(**A**) Colony formation assay was used to detect the survival curve, and Multi-target click model of GraphPad Prism 5.0 (Systat Software, USA) was used to fit cell survival curves. SKOV3: D0= 3.37,Dq=0.60, n=1.508; SKVCR: D0= 4.18, Dq=1.05, n=1.78. (**B**) SKOV3 and SKVCR cells were exposed to different radiation formulae (0Gy,1Gy×2×5,2Gy×5,10Gy×1), cell viability was analyzed by the Colony formation assay. (**C**) Dose-effects analysis in SKOV3 and SKVCR cells by CCK-8 assay. (**D**) SKOV3 and SKVCR cells were exposed to different radiation formulae (0Gy,1Gy×2×5, 2Gy×5, 10Gy×1 ), cell viability was analyzed by CCK-8 assay. *P<0.05, vs sham-irradiated.

SKOV3 and SKVCR cells were exposed to various doses of radiation (0, 2, 4, 6 or 8 Gy), cell viability was measured by Colony formation and CCK8 assay. As shown in Figure 1A and C, radiation decreased the cell viability in SKOV3 and SKVCR, the D0, Dq and N values were as follows: for SKOV3, D0= 3.37, Dq=0.60, n=1.508; for SKVCR, D0= 4.18, Dq=1.05, n=1.78. SKOV3 and SKVCR cells were than exposed to different radiation formulae(0Gy, 1Gy×2×5, 2Gy×5,10Gy×1)and cell viability was analyzed by the Colony formation and CCK8 assay. As shown in Figure 1B and D, radiation decreased the cell viability in SKOV3 and SKVCR, there was no difference among different radiation treatment. These results showed that SKOV3 and SKVCR cells exhibited differences in terms of radiosensitivity, radiation suppressed the survival fraction more significantly in SKOV3 than in SKVCR (P < 0.05).

### The endogenous expression of MAPLC3 and Caspase-3 in SKOV3 and SKVCR cells

**Figure 2:**
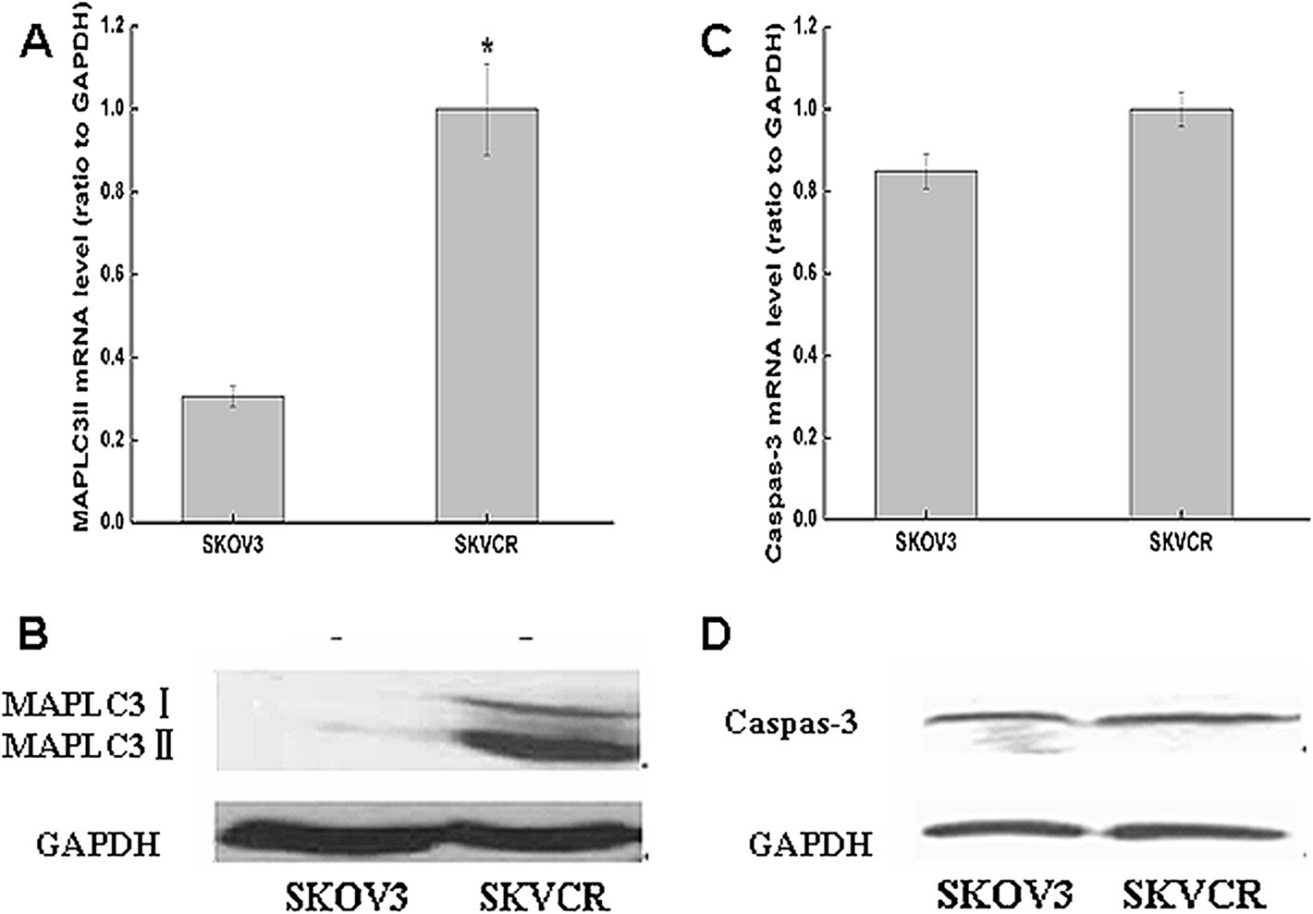
**The endogenous expression of MAPLC3 and Caspase-3 in SKOV3 and SKVCR. **(**A**) Real-time RT-PCR was used to detect the mRNA level of MAPLC3. (**B**) Western blotting was used to detect the expression of MAPLC3. (**C**) Real-time RT-PCR was used to detect mRNA level of Caspase-3. (**D**) Western blotting was used to detect the expression of Caspase-3. GAPDH was used as internal control.

After we found a variation in the radiosensitivity between the two cell lines, we sought to find the contributions of apoptosis and autophagy to the total cell death. The basal autophagic and apoptotic levels were measured, Figure 2 shown the expression level of MAPLC3 II and Caspase-3 at the mRNA and protein level which correlates with the autophagosome formation and apoptosis, respectively, the higher constitutive expression of MAPLC3 II was detected in SKVCR by real-time PCR (RT-PCR) and Western blot compared with SKOV3 cells. However, we did not find significant difference in the mRNA and protein level of both Caspase-3 and cleaved Caspase-3 (marker of apoptosis) between the two cell lines (Figure 2C and D).

### Ionizing radiation- induced apoptosis and autophagy in both ovarian cancer cells

**Figure 3:**
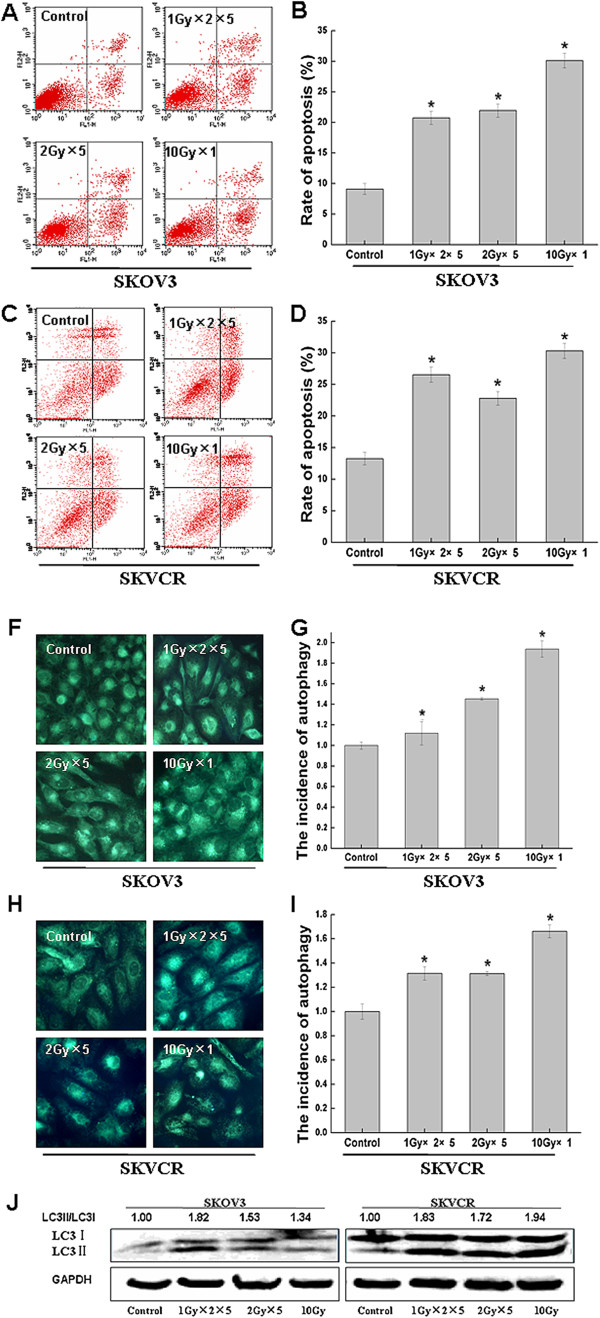
**The changes in apoptosis and autophagy after the treatment of irradiation in SKOV3 and SKVCR. **(**A**,**C**) Flow cytometry was use to quantitative the apoptotic rate in SKOV3 and SKVCR. (**B**,**D**) Statistical analysis of apoptotic rate. Results were expressed as the percentage of untreated cells, Mean ± SD of thrice. *P<0.05, vs sham-irradiated. (**F**,**H**) MDC staining was used to detect the morphologic changes of autophagy in SKOV3 and SKVCR. (**G**,**I**) Statistical analysis of autophagic rate based on MDC staining, Mean ± SD of ten vision fields. *P<0.05, vs sham-irradiated. (**J**) Western blot was used to detect MAPLC3, the increase of MAPLC3II suggested the autophagy occurrence.

To understand the underlying mechanism of radiation-induced cytotoxicity in SKOV3 and SKVCR cells, we managed to explore the roles of apoptosis and autophagy after radiation. Flow cytometric analysis was used to detect apoptosis and MDC stain and western blot were used to detect autophagy. In SKOV3 cells the basal apoptotic rate in sham-irradiated group (control) was 4.6%, exposure of cells to the different therapeutic regimens of IR significantly increased the apoptosis (Figure 3A and B). Similar scenario was exhibited in SKVCR cells (Figure 3C and D), exposure to the different regimens of IR enhanced the autophagic activity significantly in both cell lines as detected by MDC immunofluorescence and increased MAPLC3II/I ratio (Figure 3F-J).

### Enhancement of radiation-induced cytotoxicity by blocking autophagy in SKVCR cells

**Figure 4:**
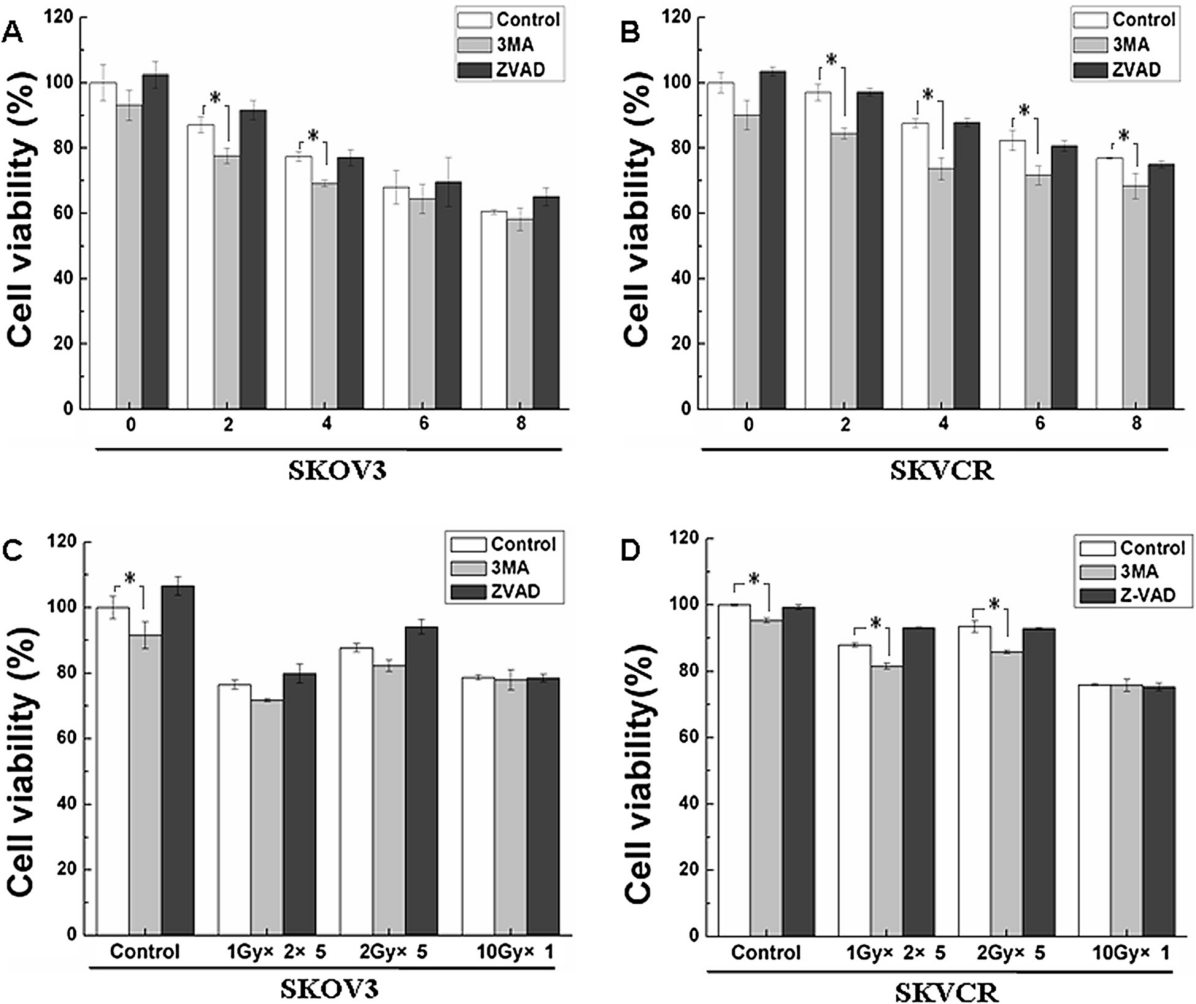
**Effects of inhibitors for autophagy and apoptosis on the cell survival rate. **(**A**) Dose-effect changes after radiation in SKVO3 cells with or without 3MA(2.5mM) and ZVAD(20μM). (**B**) Dose-effect changes after radiation in SKVCR cells with or without 3MA(2.5mM) and ZVAD(20μM). (**C**) SKOV3 cells were exposed to different radiation formulae (0Gy,1Gy×2×5,2Gy×5,10Gy×1) with or without 3MA (5mM) and ZVAD(20μM), cell viability was analyzed by CCK-8 assay. (**D**) SKVCR cells were exposed to different radiation formulae (0Gy,1Gy×2×5,2Gy×5,10Gy×1) cells with or without 3MA (5mM) and ZVAD(20μM), cell viability was analyzed by CCK-8 assay. *P<0.05, vs sham-irradiated. Results were expressed as the percentage of untreated cells, Mean ± SD of thrice. *P<0.05, vs untreated cell.

The role of autophagy during cancer therapy is paradoxical and seems to be dependent on cell context. Thus, 3MA and ZVAD were used to block autophagy and apoptosis respectively. SKOV3 and SKVCR cells were exposed to different dose of radiation (0 Gy,2 Gy,4 Gy,6 Gy,8 Gy) or different radiation formulae (0 Gy,1 Gy×2×5,2 Gy×5,10 Gy×1) with or without 3MA (2.5 mM or 5 mM)and ZVAD(20 μM), cell viability was analyzed by CCK-8 assay. As shown in (Figure 4), inhibition of autophagy significantly reduced the cell viability of both cell lines. Importantly, In SKVCR cells, 3MA significantly improved the IR-induced cytotoxicity. In SKOV3 cells the IR- induced cell death was significantly increased by 3MA in 2 Gy and 4 Gy groups (P <0.05). The IR- induced cell death was significantly increased by 3MA in SKVCR cells (P<0.05), but no change in SKOV3 cells (P>0.05). These findings indicate that autophagy plays a predominant pro-survival role in MDR-ovarian cancer cells, SKVCR.

### Transaction between autophagy and apoptosis in ovarian cancer cells

**Figure 5:**
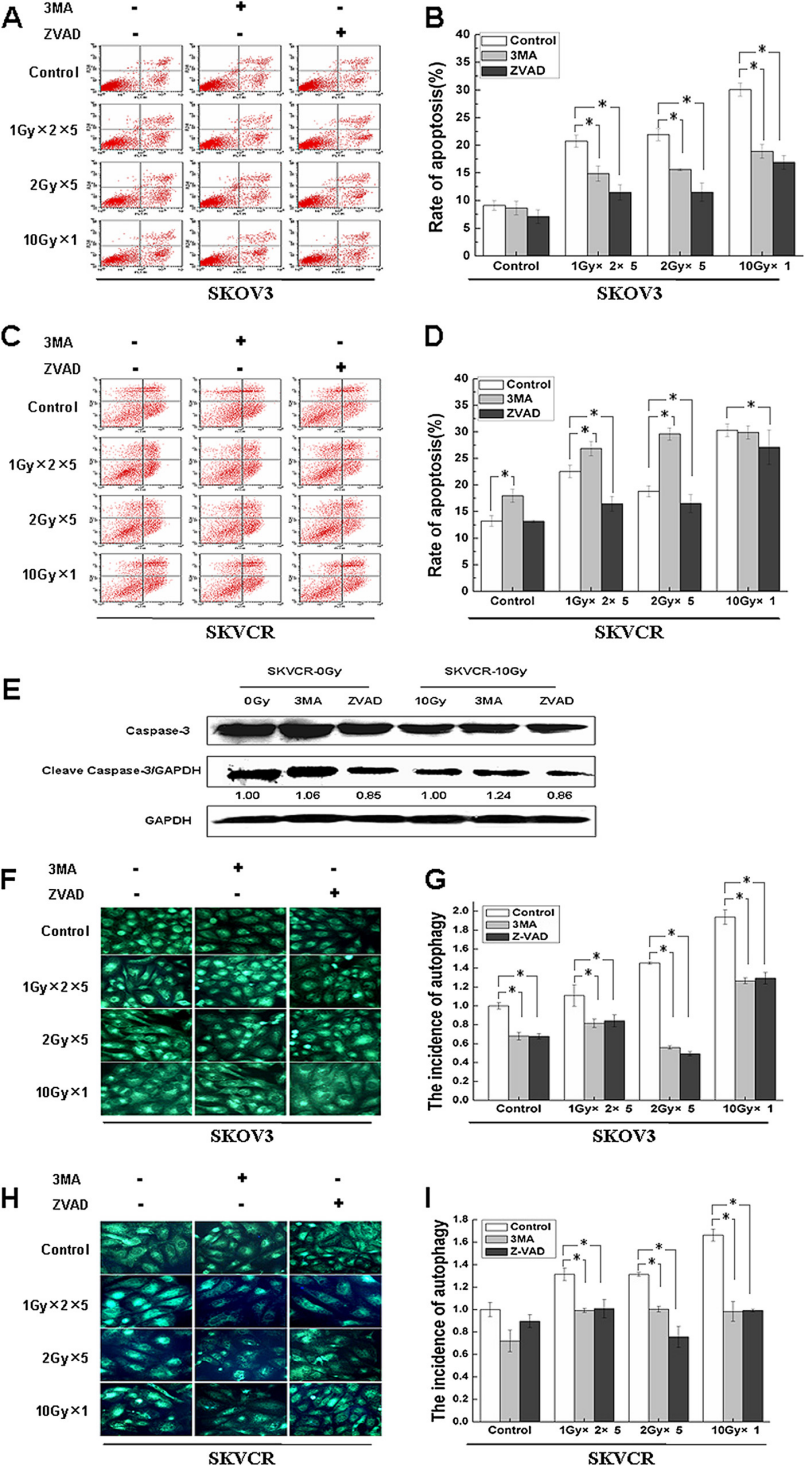
**The interrelationship between autophagy and apoptosis in SKOV3 and SKVCR.** Apoptosis and autophagy were determined in the presence or absence of 3MA or ZVAD, with or withour indicated IR regimens at 24 h. (**A**, **C**) Flow cytometry was used to quantitative the apoptotic rate in SKOV3 and SKVCR. (**B**, **D**) Statistical analysis of apoptotic rate Results were expressed as the percentage of untreated cells, Mean ± SD of thrice. *P<0.05, vs untreated cells. (**E**) Western blot was used to detect caspase 3 and cleaved caspase 3 in SKVCR, the increase of cleaved caspase 3 suggested the apoptosis occurrence. (**F**,**H**) MDC staining was used to detect the morphologic changes of autophagy in SKOV3 and SKVCR. (**G**,**I**) Statistical analysis of autophagic rate, results were expressed as the percentage of untreated cells, Mean ± SD of thrice. *P<0.05, vs untreated cells.

Next we wanted to simply delineate the underlying mechanism behind the enhanced radiosensitivity upon inhibition of autophagy. Therefore, 3MA and ZVAD were used to block autophagy and apoptosis respectively**.** Flow cytometry analysis was used to detect apoptosis, MDC stain and western blot were used to detect autophagy Pre-treatment of SKOV3 and SKVCR with ZVAD before exposure to IR markedly reduced the IR stimulatory effect on apoptosis. Pre-culture of SKOV3 cells with 3MA, significantly attenuated the IR-induced apoptosis. Importantly, in SKVCR, the apoptotic rate showed significant elevation upon pre-treatment with 3MA in irradiated and non-irradiated cells. We also found the increase of cleaved caspase-3(apoptosis marker) in SKVCR cells, wherein autophagy inhibition increased the expression of cleaved caspase-3 before and after radiation exposure (Figure 5A-E). Blocking autophagy with 3MA in SKVO3 and SKVCR significantly reduced the number of cells undergoing autophagy as estimated by MDC staining. Importantly, pre-culture of both cell lines with ZVAD attenuated the IR-induced autophagic response (Figure 5F-I). These findings indicate the presence of crosstalk between apoptosis and autophagy. More importantly, the enhanced radiosensitivity in SKVCR cells upon suppressing autophagy could be due to concomitant up-regulation of apoptosis.

### Autophagy inhibition alters the IR-induced cell cycle arrest in SKVO3 and SKVCR cells

**Figure 6:**
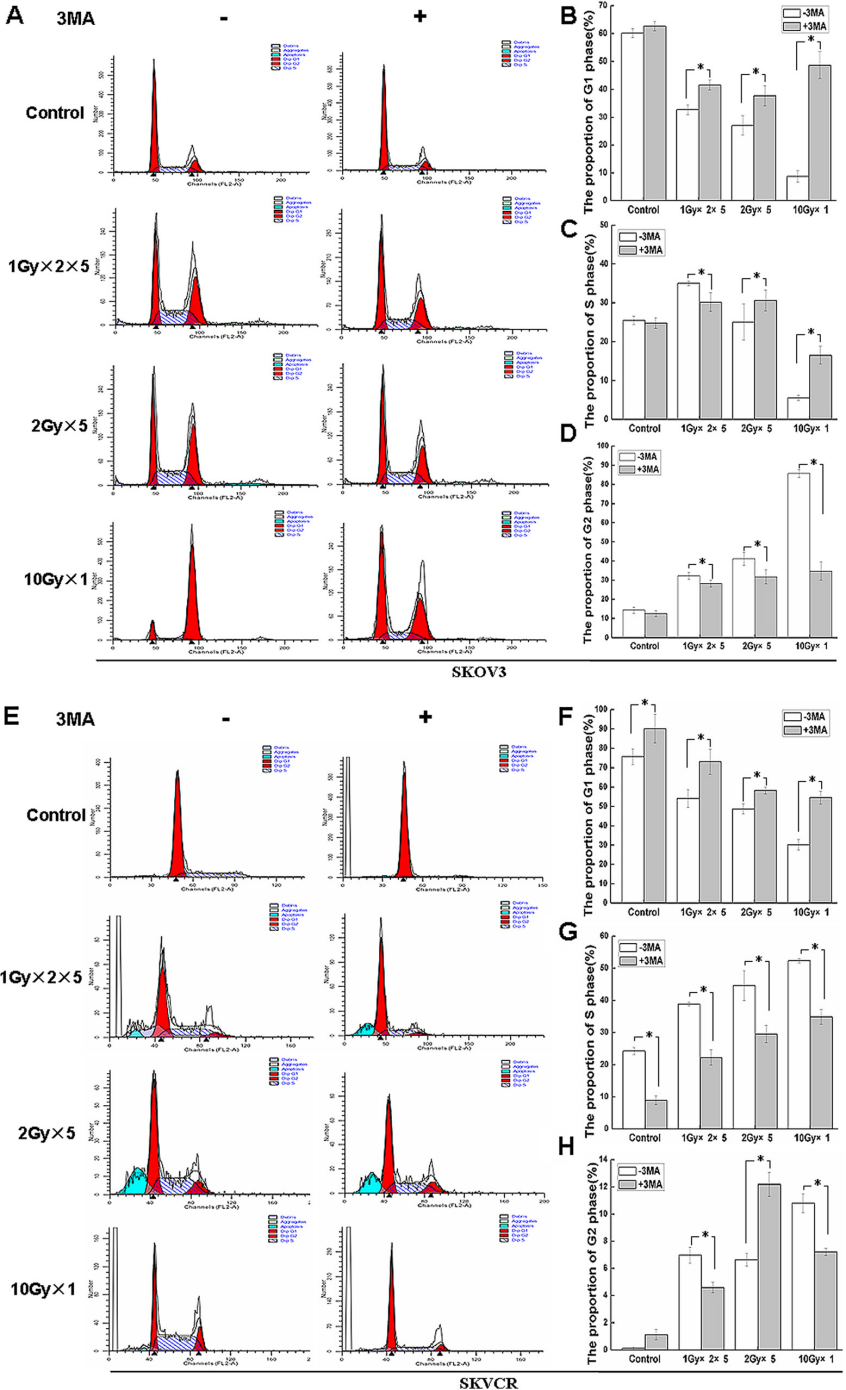
**Autophagy inhibition attenuates IR-induced cell cycle arrest. **(**A**) Flow cytometry was used to quantitative the cell cycle rate with or without autophagy inhibitor, 3MA (5 mM), with or without indicated IR regimens at 24 h in SKVO3; SKOV3 cell population (%) in G1 phase (**B**), S phase (**C**), G2 phase (**D**) were quantified. (**E**) Flow cytometry was use to quantitative the cell cycle rate with or without autophagy inhibitor, 3MA (5mM, with or without indicated IR regimens at 24 h in SKVCR. SKVCR cells were treated with IR or a combination of both (5 mM 3MA followed by IR) for 24 h, SKVCR cell population (%) in G1 phase (**F**), S phase (**G**), G2 phase (**H**) were quantified. All data are representative of three independent experiments and are shown as the mean ± SD. *P<0.05. vs untreated 3MA group.

Finally, we sought to explore whether autophagy changes was associated with cell cycle regulation. Hence, flow cytometry analysis was performed pre- and post-IR in the presence or absence of 3MA. In SKOV3 cells, radiation decreased the cell number in G1/S phase significantly and induced accumulation of cells at G2/M phase. Inhibition of autophagy in SKOV3 cells with 3MA for 24 hours prior to irradiation caused borderline ascend in G1/S phase, while G2/M phase also decreased significantly (2.5 fold), compared with IR alone group (Figure 6A-D). In SKVCR cells, radiation decreased the cell number in G1/S phase significantly and induced accumulation of cells at G2/M phase, but S phase delay was also observed. When the cells were pretreated with 3MA, the cell number in G1/S phase increased slightly, while S phase showed significant reduction after radiation as compared with radiation alone group (Figure 6E-H). These results suggest that attenuation of the IR-induced S phase delay by 3MA could be also one contributory factor in the enhanced radiosensitivity upon autophagy inhibition.

## Discussion

Ovarian cancer is one of the most common genital malignant tumors for female, maximal cytoreductive surgery together with platinum-based chemotherapy is the initial treatment program [20]. Drug resistance is a major obstacle to successful treatment of ovarian cancers [21]. Therefore, overcoming of drug-resistance in ovarian cancer would become an important factor to improve treatment efficacy. Radiotherapy functions as an adjuvant therapy for the treatment of ovarian cancer [22-24], mainly applies to the pre-operative and post-operative adjuvant therapy or palliative treatment of advanced ovarian cancer [25,26]. Radiotherapy has been shown to produce a response in chemo-resistant ovarian cancers, and may offer the possibility of improved tumor control. Radiosensitivity of malignant ovarian tumors varies markedly, dysgerminoma was classified as highly radiosensitive, while ovarian endodermal sinus tumor, emmature teratoma and embryonal carcinoma showed the lowest radiosensitivity. Epithelial ovarian cancer (EOC) and eranular cell carcinoma are moderately radiosensitive.

To improve the efficacy of treatment of drug-resistant ovarian cancer, the human epithelial ovarian cancer cell line SKOV3 and multidrug-resistant(mdr) phenotype SKVCR cells were exposed to different dose of radiation (0 Gy, 2 Gy, 4 Gy, 6 Gy, 8 Gy) or different radiation formulae (0 Gy, 1 Gy×2×5, 2 Gy×5, 10 Gy×1) with or without 3MA(autophagy Inhibitor via PI3K regulation)and ZVAD (apoptosis Inhibitor via caspase-3 regulation), the changes of cell viability, apoptosis, autophagy and cell cycle were analyzed in this study.

In order to know the radiosensitivity of ovarian cancers, cell viability was measured by colony formation and CCK8 assay, radiation could induce cell death in both cell lines and the mdr-phenotype SKVCR showed more resistant than SKVO3. In general, the radiosensitivity is proportional to the proliferation rate. One previous study found that SKOV3 and SKVCR showed different cell doubling time, the doubling time in SKVCR cells was prolonged approximately 1.8 times (53 hours for SKVCR and 29 hours for SKOV3 cells) [27]. Zhang et al. reported the mdr -transfected K-562 cells showed resistance to radiation as compared with parental cells[28], a similar trend was reported by Wei R et al. [29]. Together with our data, the fact that multi-drug resistant cells are less radiosensitive as compared with parental cells has been further confirmed. How to treat this kind of drug resistant and radiation-resistant cancer has been the hot point.

Next we managed to find what’s the difference and the underline mechanism which make SKVCR cells resistant to chemotherapy, this will contribute to find more target for treatment. It is well-known that apoptosis is important in determining the outcome of chemo- and radiation- therapy [30,31] and can be triggered by anticancer drugs and radiation. Besides, mounting evidence suggest that cancer cells can commit to death by various non-apoptotic pathways such as autophagy [32]. Then we detected the constitutive expression of different types of cell death and found that the basal level of autophagy in SKVCR was significantly higher than in SKOV3 cells, but for apoptosis there was no difference between SKVCR and SKOV3 cells, suggesting autophagy might associate with the MDR characteristics and play a pro-survival role. Autophagy is a highly conserved cellular process. It involves degradation of intracellular organelles and long-lived proteins to yield amino acids reuse and energy recycle [33]. It was demonstrated in previous researches that autophagy can have either pro-survival or pro-death functions in an organism, depending on its level of activation [34-37].

To make sure which type of cell death, autophagy or apoptosis, would play predominant roles in killing effect of radiation, 3MA and ZVAD were used to block autophagy and apoptosis respectively. Our result showed that although these two cell lines were different in terms of basal autophagy, ionizing radiation can induce both of them to undergo apoptosis and autophagy (Figure 3). Inhibition of apoptosis with ZVAD showed no impact on survival of SKOV3 and SKVCR cell lines after radiation, while inhibition of autophagy significantly decreased viability in SKVCR cells, for SKVO3 cells only low level of radiation (2 Gy and 4 Gy) could decrease the viability (Figure 4), suggesting autophagy might be the predominant mechanism after radiation rather than apoptosis.

Moreover, the complex interrelationship between apoptosis and autophagy has been reported to be affected by various biochemical processes via different pathways [38-40], and growing number of studies suggested the presence of crosstalk between autophagy and apoptosis; (a) Autophagy may be indispensable for apoptosis occurrence and lead to cell death; (b) Autophagy may antagonize apoptosis and make cells survive from stimuli; (c) Apoptosis and autophagy may occur independent of each other, there might be molecular switch between them, both autophagy and apoptosis determine the final fate of cells [41]. In this study ZVAD inhibit apoptosis and concomitantly inhibited autophagy in both cell line (Figure 5). Wu YT also got the same results and found ZVAD could inhibit lysosomal enzyme cathepsin B activity, and subsequently blocked autophagosome maturation [42]. Interestingly, 3MA play a different effect on apopotosis in SKOV3 and SKVCR, e.g., 3MA inhibit apoptosis in SKOV3, and promote apoptosis in SKVCR (Figure 5), together with inhibition of autophagy. Due to direct inhibition of autophagy and apoptosis activity by 3MA in SKOV3 cells, the pro-survival role of autophagy together with the death outcome of apoptosis attenuated the radiation-induced cell death to a significant extent. While in SKVCR cells autophagy inhibition triggered the up-regulation of apoptosis, suggesting there might be a molecular switch between autophagy and apoptosis in SKVCR cells, autophagy coordinated with apoptosis to improve the radiosensitivity. In recent years, more and more researches suggested that the apoptosis and autophagy could be mutually antagonistic or promoted in some scenario. The same induction factors can play a positive or negative role for the two kinds of programmed cell death in different cells. For example, 3MA could inhibit autophagy at the same time promote or inhibit apoptosis [43]. Which has been confirmed by our data.

In general, it has been found that cell radiosensitivity is directly proportional to the rate of cell division and inversely proportional to the degree of cell differentiation, this is described by the law of Bergonie and Tribondeau, formulated in 1906 [44]. With regard to relation between cell cycle and radiosensitivity, cells are least sensitive when arrested in the S phase, then the G1 and G2 phase, the most sensitive one is the M phase of the cell cycle. Ge JN et al. showed that starvation could not only induce autophagy in tumor cells, but also caused tumor cell cycle arrest [45]. Here, we showed that G2 / M phase arrest was induced by radiation in SKOV3 cell and SKVCR cell lines. The accumulation of G2 / M phase cells was more significant in SKOV3 cells. These results go along with many previous reports, for example, Rui Wei et al. found radiation with 6 MV X-rays caused G2/M arrest A549 and A549/DDP cells [29]. In our study, 3MA could inhibit autophagy and attenuate the radiation-induced S phase delay in SKVCR, suggesting 3MA regulate cell cycle by redistribution, adjusting radiation-induced cell cycle redistribution. Therefore, 3MA synergized the killing effects of radiation by decreasing the proportion of cells in S phase which has been thought the least sensitive phase to radiation, consequently increased the radiosensitivity of SKVCR cells.

## Conclusions

The 3MA can not only inhibit autophagy, but also inhibit the radiation- induced S phase delay and increase apoptosis in SKVCR cells, ultimately leading to the increase of cell death. These data illustrated that inhibition of autophagy may enhance the cell-killing effect of radiotherapy in multidrug-resistant human ovarian cancer cells, which may represent a novel approach to increase the efficacy of radiotherapy as an anticancer modality.

## Competing interests

The authors declare that they have no competing interests.

## Authors’ contributions

LB completed most of the experiments and drafted the manuscript. KDJ, LY, LN and HMZ performed colony formation, flowcytometry analysis and MAPLC3II detection. LXD and MSM participated importantly in the conception and design and helped to finished the manuscript. All authors read and approved the final manuscript.
